# Use of multiple failure models in injury epidemiology: a case study of arrest and intimate partner violence recidivism in Seattle, WA

**DOI:** 10.1186/s40621-019-0215-x

**Published:** 2019-08-12

**Authors:** Vivian H. Lyons, Mary A. Kernic, Ali Rowhani-Rahbar, Victoria L. Holt, Marco Carone

**Affiliations:** 1 Department of Epidemiology, School of Public Health, University of Washington, 1959 NE Pacific Street, Health Sciences Building, F-262, Box 357236, Seattle, WA 98195 USA; 2 Harborview Injury Prevention & Research Center, University of Washington, Seattle, WA USA; 3 Department of Biostatistics, School of Public Health, University of Washington, Seattle, WA USA

**Keywords:** Survival analysis, Multiple-failure survival analysis, Intimate partner violence, Injury prevention, Arrest

## Abstract

**Background:**

Single-failure survival models are commonly used in injury research. We aimed to demonstrate the application of multiple failure survival models in injury research by measuring the association between arrest and IPV recidivism.

**Methods:**

We used data from a population-based cohort of 5466 male-female couples with a police-reported, male-perpetrated incident of IPV against their female partners that occurred in Seattle, WA during 1999–2001. We estimated the risk of physical and psychological IPV recidivism (separately) for the 12 months following the index event, according to perpetrator arrest or non-arrest for the index event. We used time-dependent extended Cox regression analyses for time-to-first IPV event and Prentice, Williams and Peterson model-based analyses for time-to-multiple IPV events.

**Results:**

Arrest was associated with a reduction in time-to-first physical IPV recurrence but was not associated with time-to-first psychological IPV recurrence during the 12-month follow-up. Arrest was associated with a significantly decreased risk of physical and psychological IPV during the 12-month follow-up in the multiple failure models. The association between arrest and lower risk of physical IPV recidivism increased with increasing number of follow-up IPV events.

**Conclusions:**

We found arrest to be a plausible deterrent for recurrent IPV reduction. Our study also illustrates the use of multiple failure survival analyses in injury research. Such techniques facilitate inference about estimands that may have greater public health relevance and properly account for injury recurrence. By using multiple failure models, we were able to more deeply understand the relationship between arrest and IPV over time.

## Background

Survival analyses have gained prominence in the public health literature for situations where the outcome of interest is the time until some event of interest (e.g. cancer, cardiovascular disease diagnosis). These methods are especially valuable in injury epidemiology, where it may be of interest to explore which emergency medical interventions reduce mortality among assault patients, to determine if initiation of a drug delays incident fractures among the elderly, or to investigate how suicide risk changes following traumatic brain injury (Clark et al. 2014; Hargrove et al. 2017; Teasdale and Engberg 2001). The majority of survival analyses in injury epidemiology focus on the time to the first injury, even when recurrence is a prominent feature of injury research and the pattern of subsequent injury is of interest (Akram et al. 2016; Madden et al. 1997). We believe this indicates a missed opportunity to study injury recurrence.

While other authors have focused on describing the various survival models that allow for multiple failures, in this paper we emphasize the interpretation of the specific estimands produced by a multiple failure survival model (Amorim and Cai 2015; Ullah et al. 2014). We hope our explicit interpretations of estimands from multiple failure models and discussion of how the interpretation changes depending on the model selected will support increasing use of these models in injury epidemiology. To accomplish this, we use data from a cohort study of perpetrators of intimate partner violence (IPV) as a case study.

The study of IPV is particularly well suited for the use of multiple failure models. IPV is the use of violence, fear and/or coercion to gain power and control over one’s partner and can take the form of physical, psychological, and/or sexual abuse as well as financial abuse and reproductive control (Black et al. 2011; Breiding et al. 2014; The United States Department of Justice 2016). Although both males and females perpetrate violence against intimate partners of either sex, the most common form of IPV occurs in heterosexual relationships with a male perpetrator (Black et al. 2011). Over a third of women in the United States experience some form of IPV in their lifetime, and more than 500,000 IPV incidents are reported to the police annually (Black et al. 2011; Greenfield et al. 1998).

Survivors of IPV are at increased risk of poor health outcomes, including post-traumatic stress disorder, depression and substance abuse, much of which is believed to be secondary to the abuse they experience (Black 2011; Kernic et al. 2000; Mason and O’Rinn 2014; Coker et al. 2002). Survivors of long-term and severe abuse are more likely to experience multiple comorbidities compared to survivors of less frequent or less severe abuse (Black 2011). In 2010, the Centers for Disease Control found that among women reporting any lifetime IPV, 72.3% reported experiencing more than one type of abuse and multiple episodes of abusive events within each abusive relationship (Breiding et al. 2014). As each abusive incident increases the cumulative likelihood of injury and a multitude of other adverse health outcomes, employing successful prevention strategies that reduce IPV recidivism would likely decrease survivor injury and limit subsequent poor health outcomes. Multiple failure survival models are particularly useful to study IPV as IPV generally involves recurrent uses of specific abusive tactics, such as physical abuse, and the number, and timing of the use of these tactics matter. For this study, we used data from a cohort study conducted in Seattle, WA involving current or former partners with a police incident of intimate partner violence (IPV) that was designed to evaluate the role of arrest in physical and psychological IPV recurrence. Prior studies have attempted to assess the efficacy of secondary prevention strategies, like arrest, for individuals currently experiencing IPV, although these studies often rely on single failure models and do not consider physical and psychological IPV recurrence in their analysis. A landmark, randomized trial in Minneapolis conducted in the early 1980s was designed to examine the effect of arrest on IPV recidivism in the 6 months following an initial incident of police-reported IPV. IPV perpetrators were randomized to one of three conditions: arrest, police counseling or ordered 8 h separation between the suspect and survivor (Sherman and Berk 1984). That study found a deterrent effect of arrest on IPV recidivism, and its publication prompted the mandatory arrest laws in use today. However, there were some concerns with selective enrollment of less severe forms of abuse in the initial study. Replication studies have either found no association between arrest and recidivistic IPV or have found more conservative risk estimates, potentially due to limitations in the analysis of IPV recurrence (Berk et al. 1992; Sherman 1991; Pate and Hamilton 1992; Dunford 1990; Dunford et al. 1990; Maxwell et al. 2001). The aim of this study was to 1) describe differences in results obtained from both single and multiple failure survival methods, and 2) evaluate the utility of arrest as a secondary IPV prevention strategy in a large U.S. urban center, focusing on physical and psychological IPV recurrence perpetrated by the index perpetrator against the index survivor in the 12 months following the index incident.

## Methods

### Study sample

We used existing data from a cohort study of male perpetrators with female partners identified through the Seattle Police Department Domestic Violence Unit database. The original cohort study was designed to assess the impact of criminal justice system responses on IPV recidivism and health outcomes. Eligible incidents were those in which: (1) the Seattle Police responded to a call within the Seattle city limits during the study period of January 11,999 through December 31, 2001; (2) the call resulted in a police incident report indicating IPV in a current or former heterosexual partnership with the male partner identified as the perpetrator of abuse; and (3) only the male was identified as the perpetrator of abuse. The first police-reported IPV incident that met these criteria during the recruitment period was classified as the index incident.

A total of 6266 male/female partners met the initial inclusion criteria. We further restricted our study population to include only those with an arrestable offense at the index incident. Arrestable offenses as defined by the Washington State laws included: assault, burglary, criminal trespass, custodial interference, harassment, menacing, property damage, reckless endangerment, stalking, theft, threats and violation of court orders (Washington State Legislature 2016). Thus, we excluded 800 incidents with index offenses classified as “disturbance,” “suspicious circumstances,” “outstanding warrants” or “other offense.” The final sample consisted of 5466 former or current intimate partner couples. Perpetrator and survivor names and dates of birth were used to identify Seattle Police Department Domestic Violence Unit records for historical and recidivistic IPV incidents.

### Measures

We defined recurrent IPV, the outcome of interest, as a police-reported IPV incident between the index couple in the 12 months post-index event and classified each event as physically abusive (with or without psychologically abusive behavior) or psychologically abusive behavior only using primary and secondary offense codes for the incident. Physical IPV recurrent events were incidents with offense code(s) for assault, reckless endangerment or unlawful imprisonment. Psychological IPV only recurrent events were incidents with offense code(s) for harassment, menacing, stalking, threats, disturbance, criminal trespass, custodial interference, interfering with IPV reporting, or property damage and no recorded physical IPV offense codes. Index incidents that included sexual abuse were referred to the Seattle Police Department Sexual Assault Unit rather than the Domestic Violence Unit and were unavailable for study. Arrest of the perpetrator for the index incident served as the exposure of interest, and was obtained from police reports, as were all demographic, index event characteristics and historical IPV incident history between each index couple for the 12 months prior to the index incident. Race of the perpetrator and survivor were assessed by police officers and recorded in the incident report as either: White, African American/Black, Asian/Pacific Islander, Hispanic, American Indian/Alaskan Native or Unknown. Since racial disparities in police response have been documented in prior literature, race was included as a covariate as it may play a role in police decisions to arrest (Hepburn 1978).

### Statistical analysis

Multivariable survival analyses were used to quantify the association between arrest at the index event and the risk of police reported IPV incidents following the index incident. Arrest was coded as a time-dependent variable, with a perpetrator considered exposed only from the day of arrest onward. Analyses were conducted for the entire 12-month follow-up. Psychological and physical abuse were analyzed as separate outcomes.

We studied time to first recurrent IPV event and multiple recurrent IPV events (failures) separately. For the time to first recurrent IPV event, we used multivariable extended Cox regression analysis and allowed couples to be at risk for a first recurrence until the end of the analysis period or until there was a recurrent police-reported IPV incident (physical and psychological IPV recurrence were analyzed separately). In analyzing physical abuse outcomes, events comprising psychological abuse only were ignored rather than treated as competing events as the observation of psychological IPV does not preclude the observation of physical IPV. Models for psychological IPV likewise ignored physical IPV outcomes.

Our interest in characterizing the relative difference in the instantaneous risk of a recurrent event between subpopulations with varying number of prior IPV events suggested the use of a multiplicative intensity-based model. Alternative models have been proposed for the study of recurrent events in survival analysis, including additive regression models (Amorim and Cai 2015; Ullah et al. 2014; Schaubel et al. 2006; Cook and Lawless 2007). A model class may be deemed appropriate in the context of a given application if 1) its form is consistent with what the available science suggests about the underlying process under study (e.g. are successive events of the same type, and is their order relevant?), and 2) model coefficients can be easily interpreted on the desired scale (e.g. multiplicative versus additive contrasts) (Amorim and Cai 2015; Cook and Lawless 2007). The specific assumptions, strengths and limitations of various models available in the literature have been described in detail elsewhere (Amorim and Cai 2015; Cook and Lawless 2007; StataCorp 2015a).

Based on our knowledge and understanding of IPV perpetration, we selected the gap-time Prentice, Williams and Peterson (PWP) model as we believed IPV recidivism to be best modeled with ordered events of the same type, with time reset to zero following each event to capture the potential acceleration of IPV perpetration. The PWP model incorporates stratification based on couples’ history (number) of prior IPV events. This approach allows the baseline risk of recurrence to differ arbitrarily based on number of prior events since the index IPV event as a function of time (Prentice et al. 1982). The PWP model can be implemented using either total time, where time to each event is measured from the study baseline, irrespective of the timing of prior events, or gap time, which resets the time scale after each recurrent event. In our study, information on arrests at subsequent police-reported IPV was not available. However, had this information been available, it could have easily been incorporated into the PWP model as a time-varying covariate (e.g., as a cumulative count of arrests at prior IPV reports, or as a binary variable indicating if the latest police-reported IPV event resulted in arrest).

This model postulates that the recurrence hazard ratio (HR) comparing instantaneous risk of recurrence at any given time since the previous event in couples with and without an arrest at the index event, but otherwise similar characteristics, to be constant and common across risk set groups. This allowed the calculation of a single, overall recurrence hazard ratio (HR) for the exposure of interest.

In order to allow the HR to vary based on risk set groups defined by prior number of IPV events since baseline, we also fit a more flexible PWP model including interaction terms between arrest status and a trichotomization of prior number of events (see Appendix for statistical models used). Using this model, we estimated a HR for recurrence of IPV events separately for couples having experienced zero, one, or at least two, three or four or more follow-up IPV events. Since only 2.5% of all couples had more than four follow-up events, risk set groups were not refined further to avoid unreliable estimates. A test of null interaction in the latter PWP model suggested that these risk set group-specific HRs differed significantly. We also examined effect modification by whether the perpetrator was contacted by the police at the index incident by testing the significance of the inclusion of an interaction term between arrest and police contact. The interaction term was not significant; consequently, results are not presented separately by police contact status.

All analyses were adjusted for confounders determined a priori based on associations established in existing literature: index IPV abuse type, cohabitation, weapon use, police reported survivor injury at the index incident and police-recorded perpetrator and survivor race (white, African-American or Other) (Black et al. 2011; Hepburn 1978; Frantzen et al. 2011; Sanchez-Lorente et al. 2012; Abramsky et al. 2011; Campbell et al. 2003). Stata 14 was used for all analyses presented using the *st* suite of commands, although we additionally conducted our analyses in R to verify results (StataCorp 2015b).

## Results

### Overall cohort description

The majority of perpetrators and survivors in our cohort were either white (39.2 and 46.4%, respectively) or African-American (33.7 and 24.8%, respectively). A greater proportion of both perpetrators and survivors were between 25 and 34 years of age (34.7 and 35.6% respectively) than in other age groups. Overall, the most common relationship status in our study population at the index incident was “dating/engaged” (62.5%). Over 97% of couples in the study did not have a police reported physical or psychological IPV incident in the year prior to their index incident (partly an artifact of our inclusion criteria). Although we restricted our study population to couples with an arrestable index offense, arrest occurred in only 53.1% of the study population. By and large, this is explained by the suspect not being present when the police arrived on scene since warrantless arrest is only permitted for 4 hours following an IPV incident (Washington State Legislature 2016). Among those arrested, 86.4% were arrested within 24 h of the index incident.

### Cohort characteristics by arrest status

A greater proportion of arrested men were married to their partners than were those not arrested (25.7 and 15.1%, respectively). In incidents in which there was an arrest, it was more common that perpetrator and survivor lived together at the time of the incident than it was in those incidents without arrest (58.7 and 27.0%, respectively). Both perpetrator and survivor alcohol or drug use at the time of the incident were more likely to be noted by the responding officer in incidents with perpetrator arrest (32.5 and 14.3%, respectively) than those with no arrest (7.8 and 5.8%, respectively). Incidents leading to arrest were more likely than non-arrest incidents to involve an injury to the survivor. The most common survivor injury severity among index incidents involving an arrest was classified by the responding officer as a “minor-visible” injury (47.1%), whereas “none” was the most common injury report among index incidents involving no arrest (65.1%). Arrest-related incidents were more likely than non-arrest incidents to involve physical abuse (80.6 and 49.6%, respectively). Use of a weapon was more common among perpetrators who were arrested than those who were not (7.1 and 4.4%, respectively). Suspects in arrest-related incidents were more likely to be present at the scene when police arrived than were suspects in non-arrest incidents (87.0 and 7.8%, respectively) (Table 1) (Washington State Legislature 2016).

**Table 1:** Characteristics of the study population by arrest status at the index incident

	Not Arrested		Arrested	
	*N* = 2565		*N* = 2901	
	N	%	N	%
Perpetrator Race				
White	999	38.9	1145	39.5
Black	927	36.1	915	31.5
Asian	115	4.5	230	7.9
Hispanic	69	2.7	87	3.0
Native American	36	1.4	60	2.1
Missing	419	16.3	464	16.0
Survivor Race				
White	1183	46.1	1355	46.7
Black	688	26.8	669	23.1
Asian	178	6.9	275	9.5
Hispanic	48	1.9	55	1.9
Native American	42	1.6	75	2.6
Missing	426	16.6	472	16.3
Perpetrator Age				
18–24	457	17.8	478	16.5
25–34	853	33.3	1041	35.9
35–44	629	24.5	829	28.9
> 44	390	15.2	535	18.4
Missing	236	9.2	18	0.6
Survivor Age				
18–24	724	28.2	713	24.6
25–34	951	37.1	997	34.4
35–44	556	21.7	769	26.5
> 44	334	13.0	422	14.6
Missing	0	0.0	0	0.0
Survivor Alcohol/Drug Use at Index	149	5.8	416	14.3
Perpetrator Alcohol/Drug Use at Index	200	7.8	942	32.5
Perpetrator and Survivor lived together at Index				
Yes	692	27.0	1703	58.7
Missing	113	4.4	55	1.9
Relationship at Index				
Married	386	15.1	746	25.7
Dating/Engaged	1616	63.0	1801	62.1
Divorced	189	7.4	60	2.1
Child in Common	269	10.5	247	8.5
Separated	105	4.1	47	1.6
Survivor Pregnant at Index	57	2.22	99	3.4
Survivor Injury				
None	1669	65.1	1118	38.5
Non-visible	265	10.3	362	12.5
Minor-visible	611	23.8	1367	47.1
Severe-visible	20	0.8	54	1.9
Abuse Characteristics at Index Incident				
Physical	1272	49.6	2337	80.6
Psychological	1293	50.4	564	19.4
Weapon Used	113	4.4	205	7.1
Weapon Type^a^				
Gun	0	0.0	21	8.1
Knife	12	24.0	72	27.8
Vehicle	5	10.0	10	3.9
Other	33	66.0	156	60.2
Number of Prior Psychological IPV Events in Prior year				
0	2506	97.7	2846	98.1
1	44	1.7	42	1.5
2	9	0.4	7	0.2
3+	6	0.2	6	0.2
Number of Prior Physical IPV Events in Prior year				
0	2504	97.6	2810	96.9
1	54	2.1	72	2.5
2	5	0.2	17	0.6
3+	2	0.1	2	0.1
Suspect Contacted by Police at Index	201	7.8	2523	87.0

^a^Among those using a weapon at the index incident

### Results from time-to-event analyses

At least one police-reported physical or psychological abuse event occurred during the 12-month follow-up period for 10.3% of our cohort (562 couples). Of those with at least one police-reported abuse event, 20.5% experienced only physical IPV, 53.7% experienced only psychological IPV and 25.8% experienced both physical and psychological IPV incidents.

Arrest was associated with a 26% reduction in the instantaneous risk of a first physical IPV recurrence in the 12 months following the index incident [adjusted Hazard Ratio (aHR) = 0.74, 95% CI: 0.57–0.97]; however, arrest was not associated with a difference in risk of psychological IPV incidents during the 12-month follow-up [aHR = 1.05, 95% CI: 0.84–1.30]. Similarly, in the multiple failure analysis of all recurrences, arrest was associated with a lower risk of physical IPV recidivism [aHR = 0.79, 95% CI: 0.64–0.97], but not with a different risk of psychological IPV recidivism [aHR = 0.98, 95% CI: 0.84–1.14]. Overall, the estimated reduction in risk of IPV recidivism was not found to be substantively different when considering all recurrences rather than first recurrences alone (Table 2).

**Table 2:** Time to recurrent IPV events comparing those arrested and not arrested in Seattle, WA from 1999 to 2001

First IPV Event /Total Recurrent IPV Events		Time-to-First IPV recurrence			Time-to-Multiple IPV recurrence GAP TIME
Arrested (*n* = 2901)	Not arrested (*n* = 2565)	Crude HR (95% CI)	Adjusted HR^a^ (95% CI)	Crude HR (95% CI)	Adjusted HR^a^ (95% CI)
Physical IPV					
130 / 178	130 / 189	0.92 (0.72–1.18)	0.74 (0.57–0.97)	0.78 (0.65–0.95)	0.79 (0.64–0.97)
Psychological IPV					
221 / 410	226 / 508	0.88 (0.73–1.07)	1.05 (0.84–1.30)	0.74 (0.65–0.85)	0.98 (0.84–1.14)

Note: *CI* confidence interval, *HR* hazard ratio

^a^Adjusted for index IPV abuse type, cohabitation, weapon use, police reported survivor injury at the index incident as well as police recorded perpetrator and survivor race

In the multiple failure analysis of all recurrent events, couples in the no event since the index incident risk set had similar risk estimates for physical and psychological IPV [aHR = 0.76, 95% CI: 0.52–1.09 and aHR = 1.13, 95% CI: 0.87–1.47, respectively] as found in the adjusted single failure analysis (Tables 2 and 3). The HRs by risk set group indicated that with increasing number of preceding follow-up IPV events, arrest was associated with a greater reduction in risk of recurrent IPV for both physical and psychological IPV.

**Table 3:** Time to recurrent IPV events comparing those arrested and not arrested by risk set in Seattle, WA from 1999 to 2001

	Time-to-Multiple IPV recurrence Adjusted HR^a^ (95% CI)
Physical IPV	
No events since baseline	0.76 (0.52–1.09)
One event since baseline	0.85 (0.57–1.27)
Two events since baseline	0.59 (0.33–1.06)
Three events since baseline	0.59 (0.25–1.36)
Four or more events since baseline	0.50 (0.21–1.18)
Psychological IPV	
No events since baseline	1.13 (0.87–1.47)
One event since baseline	0.80 (0.61–1.05)
Two events since baseline	0.73 (0.52–1.03)
Three events since baseline	0.59 (0.39–0.91)
Four or more events since baseline	0.59 (0.33–1.06)

Note: *CI* confidence interval, *HR* hazard ratio

^a^ Adjusted for index IPV abuse type, cohabitation, weapon use, police reported survivor injury at the index incident as well as police recorded perpetrator and survivor race

## Discussion

This study demonstrated a significantly reduced risk of recurrent physical IPV in both the single and multiple failure analyses in the 12 months following an index episode of IPV among heterosexual couples in which the male suspect was arrested compared to couples in which the male suspect was not arrested. The difference observed between HRs in the time-to-multiple physical IPV events analysis stratified by number of prior IPV events may incorporate the potential downstream criminal justice response to recurrent IPV events, compared to a single IPV event. If a couple has multiple physical IPV events, the perpetrator may be more likely to be arrested and face charges for subsequent IPV events than for the initial IPV event.

Our study not only deepens our understanding of the association between arrest and IPV, but it allows a critical assessment of the use of multiple failure models in injury research. While the estimands produced by the time-to-first event and time-to-multiple events analyses are similar, their interpretations differ qualitatively. The estimand resulting from the time-to-first event analysis based on the proportional hazards model pertains to the relative instantaneous risk of a first reported IPV event since the index event comparing those arrested at the index event to those not. The estimand in the time-to-multiple events analysis based on the gap-time PWP model without risk group interaction quantifies the overall relative risk of a subsequent IPV event among couples with the same history of IPV recurrence (same number of prior IPV events and time elapsed since last event) comparing those arrested at the index event to those who were not. The time-to-multiple events analysis allows for a varying HR by risk set group, producing estimands with a more focused interpretation based on number of prior IPV events experienced. Although the values of the estimands may be similar between these survival analyses, this may be an artifact of the timing of IPV recurrence in our study, as multiple failure models account not only for the number of subsequent events but also for their timing relative to the index event. Another study may find a larger difference between estimands produced by single and multiple failure analyses.

Additionally, a multiple failure analysis provides estimands that may be of greater interest than those produced by a single failure analysis and provide additional understanding into the relationship between the exposure and outcome. The HR produced by the gap-time PWP model (without risk group interactions) estimates the overall association between arrest and IPV recidivism, arguably the true relationship of interest as arrest often does not result in jail or prison, allowing additional IPV perpetration following an arrest. A multiple failure analysis by risk set group allowed us to find evidence of a differential association between arrest and instantaneous risk of IPV recidivism as a function of the number of prior IPV events, something not observable in single failure analyses.

Besides providing insight into the use of multiple failure survival analyses in injury research, this study adds to the body of literature that investigates the association between arrest and IPV recidivism. Our findings support the initial study that prompted the mandatory arrest laws. Results from the initial randomized trial by Sherman et al. assessing the impact of arrest on IPV recidivism in Minneapolis indicated a 62% reduction in police-reported IPV recurrence and a 55% reduction in survivor-reported IPV recurrence among perpetrators arrested compared to perpetrators who received counseling from police officers (Sherman and Berk 1984). However, the investigators noted concerns of police officers differentially enrolling couples based on perceived severity of abuse, potentially restricting their study findings to a group of perpetrators with less severe forms of abuse.

Following this seminal study, five replication studies were completed to attempt to reproduce these findings in other cities. Three of these studies found no association between arrest and IPV recurrence; two others found some evidence supporting a deterrent effect of arrest, but only among employed suspects or using survivor interviews for IPV recurrence ascertainment (Sherman and Berk 1984; Pate and Hamilton 1992; Dunford 1990; Dunford et al. 1990; Berk and Newton 1985; Sherman et al. 1992; Hirschel et al. 1992). These conflicting results have prompted much debate about the role formal and informal sanctions play in deterring IPV and the potential effect modification observed among suspects with a greater stake in conformity (i.e. employed, married). It is also possible that the consequences of arrest are not a sufficient deterrent for a higher risk group of IPV perpetrators. The lack of consistent replication highlighted the need for further study of this important issue.

Although this study was not a randomized controlled trial, we believe its findings can nevertheless inform policy and practice. Unlike the randomized controlled trials and meta-analysis, this study is the first of its size to assess the effectiveness of arrest on IPV recidivism deterrence at a population level. As this study did not randomly allocate arrest, and utilized police-records, we were able to enroll eligible couples with severe forms of abuse that would have been excluded in the other studies. In our study population, it was equally likely that perpetrators would be arrested for an arrestable offense as not, maximizing our power and ability to measure precisely the association between arrest and IPV recurrence. We controlled for characteristics likely to play a role in whether a perpetrator was arrested at the index incident, including abuse type, alcohol and drug use, weapon use, survivor race, survivor injury and cohabitation. Additionally, this study took place after the implementation of mandatory arrest laws in Seattle prompted by the findings of Sherman et al. and sheds light on the impact of the real-world implementation of these laws. Finally, this study highlights the application of time-to-first and time-to-multiple IPV events models for injury research. We believe that in this study, the time-to-multiple IPV events models are the most salient since the ultimate objective is to not only reduce the occurrence of a first IPV recurrence, but also of serial IPV recidivism.

We found arrest to be associated with a decreased risk in physical IPV recurrence but found no similar statistically significant association with psychological IPV recurrence. This may be a reflection of psychological IPV being less straightforward in its definition, presenting evidence and meeting evidentiary standards. While overall 53.1% of perpetrators were arrested in response to their index incident, a greater proportion of perpetrators of physical IPV at the index incident were arrested compared to perpetrators of psychological IPV (64.8% vs 30.4%, respectively). This difference in proportion arrested likely reflects, at least in part, the challenge police face in determining whether or not a psychological IPV event is an arrestable offense. In addition, the psychological IPV category reflects a larger, more heterogeneous group of behaviors than physical IPV. It is possible that arrest is a successful deterrent of the particular psychological IPV behavior that led to an arrest, but not a deterrent to other forms of psychological IPV.

Our study should be considered in light of its limitations. First, it is possible that we missed IPV events between study couples during the follow-up period. While this could occur in the presence of substantial migration of survivors and/or perpetrators out of the Seattle area and hence out of the Seattle Police Department’s jurisdiction, the results of the study by Holt et al. from the same geographic area during the same time period are reassuring. In that study the authors were able to maintain contact for 12 months with 83% of women with a protection order (PO) and 74% of those without a PO (Holt et al. 2003). We have no basis to suspect a more extreme pattern of migration in our study or differential migration by arrest status. We were limited in our study to police-reported IPV incidents, missing the portion of recurrent IPV events that were not reported to police. If IPV survivors are motivated to prevent police involvement due to concerns that it may result in another arrest of their partners, differential reporting by arrest status may result. While we could not measure this directly, we are reassured that neither the study by Sherman et al. nor the subsequent replication studies found such differential reporting to occur (Sherman and Berk 1984; Dunford et al. 1990).

This study was also limited to the information that was available from police records for the events that we captured. One variable of interest from prior studies is perpetrator employment status; arrest has been shown to have a greater deterrent effect among employed men (Pate and Hamilton 1992). If the effect modification found in the studies by Pate et al. and by Berk et al. were to hold true in our study, it is possible that the hazard ratios for this subset of our study cohort would have been even lower. We also did not have data on whether arrests for the index IPV event led to incarceration. However, since arrest may trigger a number of different criminal justice system responses that would lie on the causal pathway to subsequent IPV, we believe the association between arrest and lower risk of IPV recidivism is important whether or not the underlying reason for the lower rates is due to arrest itself or subsequent incarceration. Additionally, a nationally representative survey found that fewer than 8% of perpetrators of IPV were sentenced to jail or prison when the abuse was known to police (Hamby et al. 2015). Likely, few of the perpetrators in our study spent time incarcerated or were deterred primarily due to incarceration. We were also unable to consider death of study participants during the follow-up period, which would have precluded further IPV recidivism within the couple. Finally, there may be uncontrolled confounding due to the quality of data available in police records, as is typical for this type of observational study. However, the magnitude of observed risk is likely not entirely explained by this type of uncontrolled confounding and suggests a real association between arrest and reduction of IPV perpetration.

## Conclusions

Importantly, this study provides insight into the implementation of arrest in practice following adoption of mandatory arrest laws. According to our results, arrest may be a plausible deterrent for recurrent physical IPV reduction. Future studies should aim to understand the mechanisms that support a decrease in risk of IPV recurrence following arrest in order to better focus prevention and intervention efforts. For instance, arresting a higher proportion of suspects by expanding the timeframe for arrest following an IPV event and enforcing mandatory arrest laws may be found to reduce IPV recurrence. Although arrest does not prevent all future IPV, any reduction in IPV events provides an important benefit in terms of public health. Finally, we believe that investigators interested in conducting injury research on outcomes where recurrence is an important component of the theoretical model (e.g. abuse, traumatic brain injury) should consider using a multiple failure survival analysis in order to provide a more comprehensive and more interpretable assessment of the association between relevant outcomes and exposures.

## Data Availability

Deidentified data are available upon request.

## Appendix

### Form of Regression Models Implemented in the Statistical Analysis

Below, *λ(t|h(t))* refers to the hazard of a first IPV recurrence (in time-to-first event analyses) or the intensity of IPV recurrence (in time-to-multiple events analyses) at time *t* as a function of the available history *h(t)*. The history *h(t)* consists of all information recorded up to time *t*, including baseline covariate information, information on arrest time related to the index event (if applicable), and history of IPV events since the index event. The vector of baseline covariates is denoted by *z*, whereas *x(t)* is an indicator of having experienced arrest (as a consequence of the index event) by time *t*. The symbol *n(t)* refers to the number of IPV events experienced after the index event by time *t*, whereas at any time *t*, *τ(t)* is the time at which the last IPV event occurred. For any natural number *m≥0*, *λ*_*m*_ refers to the baseline hazard (or intensity) function for couples having experienced exactly *m* recurrent IPV events since the index event, and *β*_*m*_ is the regression coefficient associated to arrest pertaining to couples with this same number of prior IPV events.

Time-to-first event Cox model:

$$ \uplambda \left(t|h(t)\right)={\uplambda}_0(t)\exp \left\{{\alpha}^Tz+\beta x(t)\right\} $$

Time-to-multiple events gap-time PWP model with common arrest exposure coefficient:

$$ \uplambda \left(t|h(t)\right)={\uplambda}_{n(t)}\left(t-\tau (t)\right)\exp \left\{{\alpha}^Tz+\beta x(t)\right\} $$

Time-to-multiple events gap-time PWP model with history-specific arrest exposure coefficients:

$$ \uplambda \left(t|h(t)\right)={\uplambda}_{n(t)}\left(t-\tau (t)\right)\exp \left\{{\alpha}^Tz+{\beta}_{n(t)}x(t)\right\} $$

## Abbreviations

HR: Hazard Ratio
IPV: Intimate Partner Violence
PO: Protection Order
PWP: Prentice, Williams and Peterson Model
